# Involvement of Multiple Ion Channels and Receptors in Mediating the Insecticidal and Repellent Actions of Limonene

**DOI:** 10.3390/ijms27010416

**Published:** 2025-12-30

**Authors:** Yuan Li, Wilson Valbon, Felipe Andreazza, Ke Dong

**Affiliations:** Department of Biology, Duke University, Durham, NC 27708, USA; yuan.li@duke.edu (Y.L.); wilson.valbon@gmail.com (W.V.)

**Keywords:** yellow fever mosquito, odorant receptors, TRPA1 proteins, Rdl, positive allosteric modulators

## Abstract

R-limonene has been integrated into various pest control practices as a repellent or an insecticide. However, how limonene induces aversion or mortality remains largely unknown. To elucidate the underlying mechanisms, we conducted behavioral, toxicological, and electrophysiological assays in *Aedes aegypti*, a primary vector of human diseases. To investigate whether limonene acts on voltage-gated sodium channels and/or the Rdl (Resistance to dieldrin) receptor, two major targets of neuroactive insecticides, we characterized the effect of limonene on *Ae. aegypti* sodium and Rdl channels expressed in *Xenopus* oocytes. Limonene significantly potentiated GABA-induced chloride currents through Rdl in a concentration-dependent manner but had no effect on sodium channels. For repellency, limonene evoked spatial repellency in wild-type mosquitoes; however, the spatial repellency by limonene was significantly reduced in knockout mutants of *Orco^−/−^* (odorant receptor co-receptor) and *TRPA1^−/−^* (Transient Receptor Protein, subfamily A and member 1). These results indicate that limonene likely targets the Rdl receptor for insecticidal activity and limonene spatial repellency requires both Orco and TRPA1 channels. Our results reveal the involvement of multiple ion channels and receptors in the mosquito nervous system for limonene’s insecticidal and/or spatial repellency actions, highlighting limonene’s potential as a multi-target neuroactive agent for pest control.

## 1. Introduction

(R)-limonene, hereafter referred to as limonene, is a naturally occurring monoterpenoid that is abundantly found in the essential oil extracts of citrus fruits [1]. The versatile property and the pleasant lemon-like fragrance of limonene make it a widely used valuable ingredient in cosmetic formulations and food products [1]. Additionally, limonene is recognized for its anti-inflammatory and antioxidant effects and has been commonly incorporated into therapeutic applications [2,3]. Besides the functions mentioned above, limonene is one of the first few naturally derived compounds registered as insecticides in the US [1]. Previous studies have demonstrated that limonene exerts insecticidal and/or repellent effects against a range of veterinary, urban, and agricultural pests, including horn flies, bed bugs, scale insects, and mealybugs [4,5,6]. Notably, pollinators such as honeybees (*Apis mellifera*) and bumble bees (*Bombus impatiens*) are attracted to limonene without being harmed [7,8].

Disease vector mosquitoes are among the deadliest insects to human life, threatening hundreds of millions of lives by transmitting arboviruses and parasites [9,10]. The rising resistance of mosquito populations toward currently used insecticides is of great concern and highlights the need for alternative management strategies [10,11]. Studies conducted over the past decades have illustrated the effectiveness of limonene as a mosquito control agent. For instance, limonene is a potent larvicide against multiple mosquito species including *Aedes aegypti*, *Anopheles stephensi*, and *Culex quinquefasciatus* [12,13,14]. Moreover, Hebeish et al. evaluated how adult female *Ae. aegypti* responded to cotton fabrics coated with limonene and categorized the observed effects into repellency, knockdown, and mortality [15]. The potent repellency effect of limonene was further supported by Nematollahi et al. in a two-choice behavioral assay without host cues, and by Nascimento et al. in a hand-in-cage bioassay showing dose-dependent deterrence in the presence of host cues [12,16]. Collectively, these studies demonstrated limonene as a promising candidate for the development of mosquito control strategies. However, no information is available regarding the molecular mechanisms that underly the toxicity and repellency effects of this compound on mosquitoes or any other insect species.

Ion channels are of crucial importance in the insect nervous system function and are targets of neuroactive insecticides, including essential oil components. For instance, voltage-gated sodium channels (Na_V_), which are essential for the initiation and propagation of action potentials, have been shown to be targeted by pyrethrum, the dried flower extracts from *Chrysanthemum* [17]. Gamma-aminobutyric acid (GABA) receptors are ligand-gated chloride channels mediating inhibitory synaptic transmission. A previous study has shown that the “resistance to dieldrin” (Rdl) receptor, a major insect GABA receptor, of *Ae. aegypti* is targeted by nootkatone in vitro, which potentially contributes to the insecticidal and/or repellent effects of this compound [18].

Odorant receptors (Ors) are a family of chemoreceptor proteins that are primarily responsible for detecting volatile compounds in insects [19,20]. These proteins form heteromeric ion channel complexes composed of a ligand-specific subunit and an obligatory co-receptor subunit [19]. Ors have been frequently implicated in mediating the repellency effects of essential oil components against mosquitoes and other insects. For example, a rose-scented compound, 2-phenylethanol, selectively activates CquiOr4 in *Cx. quinquefasciatus* and triggers strong avoidance behaviors [21]. AaOr31 is activated by E-(β)-farnesene (EBF), which is present as a minor component in pyrethrum; and AaOr31 activation by EBF contributes significantly to pyrethrum spatial repellency in *Ae. aegypti* [17]. Similarly, Or49, another Or with conserved expression in the maxillary palps of *Culicine* mosquitoes, is responsible for mediating spatial repellency against (+)-borneol, a volatile terpene found in camphor trees [22]. In mosquitoes, a single gene encodes the co-receptor of Ors (Orco), and deprivation of Orco expression leads to impaired Or-mediated olfactory response [23]. Consistent with this, spatial repellency by pyrethrum and nootkatone was significantly reduced in a mosquito knockout line lacking Orco expression [17,18].

In addition to olfactory receptors, transient receptor potential channels (TRPs), a group of multifunctional sensor that respond to thermal, chemical, and noxious reactive compounds, also play a role in mediating mosquito repellency and antifeeding [24,25,26,27,28,29,30]. Cinnamodial, a sesquiterpenoid derived from the bark of *Cinnamosma fragrans*, exerts antifeedant effects against *Ae. aegypti* during both sugar and blood feeding, and this effect is significantly reduced in a *TRPA1*-deficient line [29]. Similarly, nepetalactone, a monoterpenoid massively found in catnip, functions as a feeding deterrent against *Ae. aegypti* in a membrane feeder bioassay, and this antifeedant effect is also abolished in *TRPA1^−/−^* mosquitoes [28].

Given the broad repertoire of molecular targets essential oil compounds can potentially target, we took behavioral, toxicological and electrophysiological approaches to evaluate multiple ion channels and receptors for their possible involvement in the action of limonene as an insecticide and a repellent against the yellow fever mosquito *Ae. aegypti*.

## 2. Results

### 2.1. Limonene Potentiates GABA-Dependent Chloride Currents of AaegRdl1-1 In Vitro

We first investigated the mechanism of insecticidal action of limonene by directly examining the effects of limonene on ion channels. Our lab has established *Xenopus* oocyte-based heterologous expression system to directly examine the effect of neurotoxins on voltage-gated sodium (Na_V_) channels and Rdl GABA-gated chloride channels, which are major molecular targets of several neuroactive insecticides. We started by testing oocytes expressing the *AaNa_V_1-1* channel with a series of protocols to explore the possible effects of limonene on Na_V_ channel gating properties. The results showed that application of 100 µM limonene did not alter the voltage dependency of Na_V_ channel activation or inactivation (Supplementary Figure S1A–C). We subsequently evaluated whether limonene acts similarly as pyrethroids and elicits any non-inactivation current and/or tail current of Na_V_ channels. No obvious differences were observed in the recording traces of Na_V_ currents before and after 100 µM limonene application (Supplementary Figure S1D,E). Taken together, our results indicate that AaNa_V_ is unlikely to be a molecular target of limonene.

**Figure 1:**
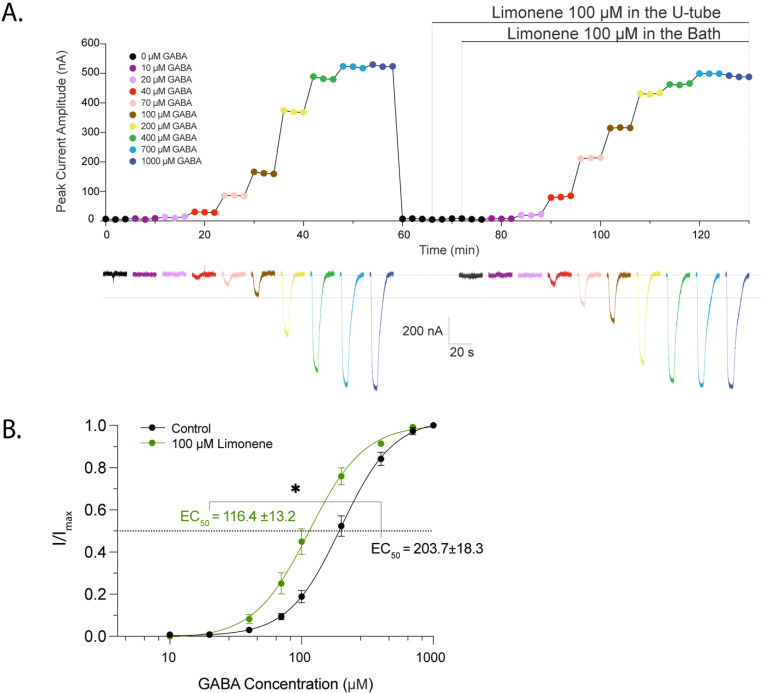
Limonene enhances GABA-induced current of *AaegRdl1-1* expressed in *Xenopus* oocytes. (**A**) Time course of peak current amplitudes of a representative Rdl-expressed *Xenopus* oocyte in response to pulses of GABA at concentrations ranging from 0 to 1000 μM in absence or presence of limonene. Oocyte was clamped at −80 mV. Co-application of 100 μM limonene initiated at 66 min in the U-tube and at 72 min in the bath. Each GABA/limonene pulse was repeated for three times at 2 min intervals to access steady state equilibrium on the peak current response before testing the next GABA concentration. Representative recording traces of corresponding treatments are presented at the bottom of the panel. (**B**) Dose–response curves of Rdl to GABA application, with (green) and without (black; control) the co-application of 100 μM limonene. Current responses were normalized to the maximum currents induced by 1000 μM GABA alone (black), or 1000 μM GABA plus 100 μM limonene (green) within each oocyte. Scatter plots are mean ± s.e.m; n = 5 replicates, and continuous line is the global fitted curve. See methods for details. Asterisk indicates significant difference as determined by Student’s *t*-test (*p* = 0.005) on the EC_50_ values.

We next examined the effect of limonene on the *AaegRdl1-1* channel expressed in oocytes. Stepwise increasing concentrations of GABA were briefly pulsed onto oocytes by a U-tube system in the recording chamber every 2 min, eliciting dose-dependent inward chloride currents (*I_GABA_*) (Figure 1A). The amplitude of peak *I_GABA_* plateaued with 1000 µM GABA application with an average GABA EC_50_ at 203.7 ± 18.3 µM (n = 5; Figure 1B). *I_GABA_* was blocked by the application of a positive control, fipronil, as expected (Supplementary Figure S2). Application of 100 µM limonene, either as pulses via the U-tube or constant presence in the bath solution did not trigger any currents (Figure 1A), suggesting that limonene is not a direct agonist of mosquito GABA receptor. We then co-applied 100 µM limonene with stepwise increasing concentrations of GABA. As shown in the representative traces, limonene effect was evident across a broad range of GABA concentration (Figure 1A). Consistently, the analysis of *AaegRdl1-1* response curve revealed that application of 100 µM limonene shifted the relationship leftward, resulting in a GABA EC_50_ that is significantly smaller than that observed without co-application of limonene (Figure 1B).

**Figure 2:**
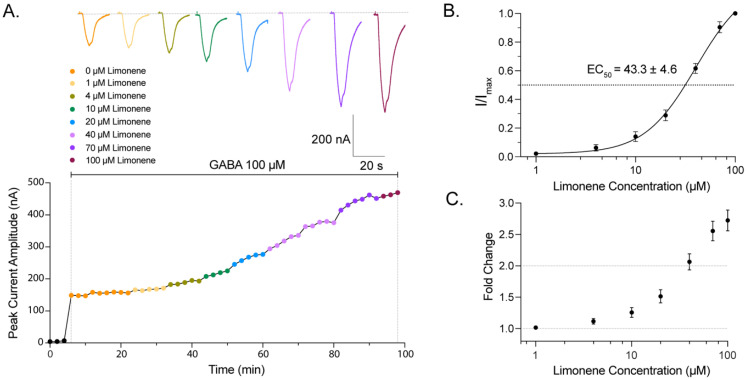
Limonene potentiates GABA-induced current of *AaegRdl1-1* expressed in *Xenopus* oocytes in a dose-dependent manner. (**A**) Peak current amplitude of a representative Rdl-expressing *Xenopus* oocyte in response to limonene concentrations ranging from 0 to 100 μM over a 98 min recording period. Oocytes were clamped at −80 mV. Application of 100 μM GABA pulses initiated at 6 min, and co-application of limonene in the bath and in the U-tube began at 24 min. GABA pulses at the same limonene concentration were repeated for at least three times or until effect become stable. Representative recording traces of corresponding treatments are presented at the top of the panel. (**B**) Dose response curve of Rdl to limonene with the co-application of 100 μM GABA pulses from the U-tube. Current responses are normalized to the maximum currents. Scatter plots are mean ± s.e.m, n = 5 replicates; and continuous line is the global fitted curve. (**C**) Fold change in limonene-induced current amplitude with the co-application of 100 μM GABA relative to the control current induced by 100 μM GABA alone. Values are mean ± s.e.m; n = 5 replicates.

We subsequently used U-tube pulses of a fixed GABA concentration (100 µM) to establish a response curve of *AaegRdl1-1* to stepwise increases in the concentration of limonene (Figure 2A). Peak current amplitude of *I_GABA_* increased in a limonene-concentration dependent manner, with an average limonene EC_50_ of 43.3 ± 4.6 µM (n = 5; Figure 2B). With 100 µM limonene application, the peak current amplitude was enhanced by 2.7 ± 0.2 folds relative to the application of 100 µM GABA alone (Figure 2C).

### 2.2. Limonene Repellency Is Dependent on Orco and TRPA1 Channels

**Figure 3:**
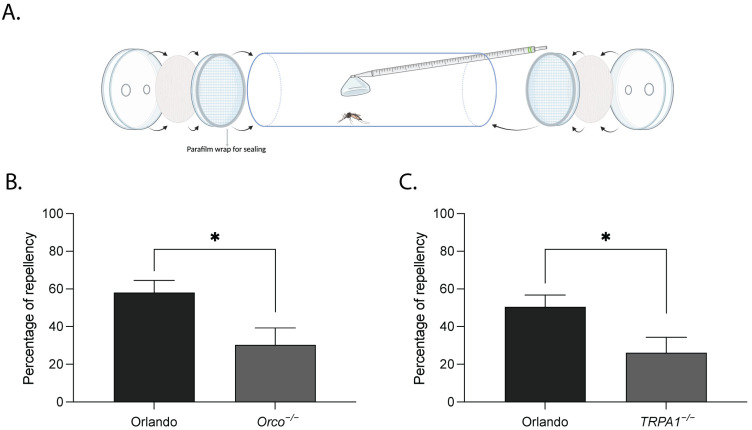
Limonene spatial repellency is dependent on Orco and TRPA1 channels. (**A**) Experimental setup of the two-choice spatial repellency tube assay. Illustration created in BioRender. (**B**,**C**) Percentages of repellency in *Orco^−/−^* (**B**) and *TRPA1^ECFP1/ECFP2^* (**C**) with the corresponding wildtype Orlando as control. For each mutant line, three independent batches were tested for comparisons with the wildtype. Six replicates were performed per batch on the same day, yielding n = 18 for statistical analysis. Values are mean ± s.e.m. Asterisks indicate significant difference between mutant and wildtype lines as determined by Student’s *t*-tests (*p* < 0.05).

To evaluate the activity of limonene as a mosquito repellent, we first used a hand-in-cage bioassay evaluating spatial repellency in the presence of host cues. We found that the average landing numbers of mosquitoes significantly decreased after the application of limonene, reaching repellency percentages of 70.9 ± 4.0 (n = 5) and 96.6 ± 0.5 (n = 7), respectively, at 12 µg/cm^2^ and 120 µg/cm^2^ in wildtype Orlando mosquitoes (Supplementary Figure S3). To avoid a confounding indirect effect of limonene inhibition of host attraction on the observed avoidance behavior, we conducted a tube assay in the absence of host cues (Figure 3A), which directly evaluates spatial repellency evoked by limonene. Similarly, we observed limonene repellency in wildtype Orlando mosquitoes, reaching a repellency percentage of 54.3 ± 4.5 (n = 36; Figure 3B,C) in response to limonene applied at 4.2 mg per filter paper. To evaluate whether Orco- and TRPA1-mediated chemosensory pathways are involved in limonene repellency, we ran the tube assay using two mutant lines of *Ae. aegypti*: *Orco^−/−^* and *TRPA1^−/−^*. The *Orco^−/−^* line lacks functional olfactory signaling mediated by Ors due to a disrupted co-receptor gene, and *TRPA1^−/−^* line carries a loss-of-function mutation of TRPA1 protein. Both *Orco^−/−^* and *TRPA1^−/−^* mosquitoes were significantly less repelled by limonene, with an average repellency percentage at 30.3 ± 9.0 (n = 18; Figure 3B) and 26.2 ± 8.2 (n = 18; Figure 3C), respectively.

### 2.3. Mosquito Knockdown by Limonene Is Independent of the TRPA1 Channel

**Figure 4:**
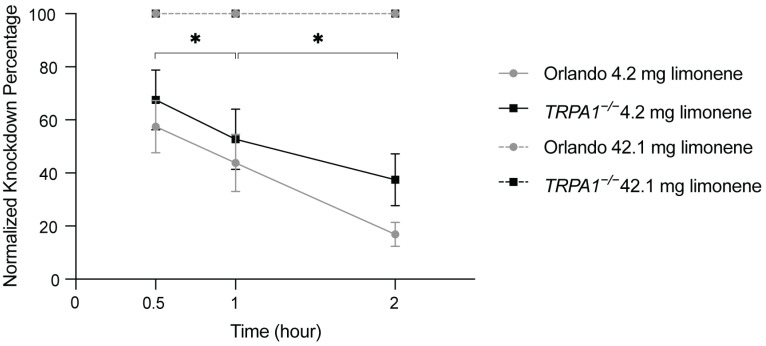
TRPA1 channel is not involved in limonene evoked vapor toxicity against female *Ae. aegypti.* Figure presents percentage of knockdown of Orlando (gray) and TRPA1^−/−^ (black) at 30 min, 1 h, and 2 h post limonene exposure. Data are mean ± s.e.m. Percentage of knockdown significantly increased over time (F_(2,28)_ = 37.56, *p* < 0.001), as determined by an RM two-way ANOVA. Asterisks indicate significant differences between time points, as determined by Fisher LSD post hoc tests. Genotype had no significant effect. Replicates for 42.1 mg per filter paper limonene application had no variance among technical replicates; therefore, no error bars are shown.

Given the broad expression of TRPA1 channels in the insect nervous system, as previously reported in *D. melanogaster* [31], we investigated whether the TRPA1 channel, in addition to be required for limonene repellency, is also required for limonene insecticidal activity. To address that, we carried out a Petri-dish based vapor toxicity assay (Supplementary Figure S4) to compare the susceptibility between Orlando and *TRPA1^−/−^* lines in response to limonene. The results showed that both lines presented impaired mobility within 30 min after exposure to limonene application at 4.2 mg per filter paper and then gradually recovered from paralysis within 1–2 h (Figure 4). Nevertheless, no significant difference was found between wildtype and *TRPA1^−/−^* lines at any time point post-exposure (F_(1,28)_ = 1.038, *p* = 0.326). Exposure to a 10-fold higher concentration of limonene caused complete (100%) knockdown in all wildtype and *TRPA1^−/−^* mosquitoes within 30 min post-exposure, and neither line subsequently recovered from the paralysis.

## 3. Discussion

Our study confirmed that limonene exerts spatial repellency at sublethal concentrations and vapor toxicity at higher concentrations against *Ae. aegypti* mosquitoes. Notably, our findings revealed the involvement of multiple channels and receptors in limonene-induced repellency, contributing to the growing list of natural compounds that evoke spatial repellency through Or-dependent and/or Or-independent mechanisms. In addition, we also found that limonene potentiates the inhibitory action of GABA on Rdl receptors, a major target of neuroactive insecticides. These findings provided new information on understanding of the molecular mechanisms underlying spatial repellency and toxicity of natural plant compounds and highlight the potential of targeting diverse neural pathways for vector control strategies.

The involvement of Orco in limonene repellency against mosquitoes is not unexpected, as a previous study reported that limonene activates specific olfactory neurons in the antenna and elicits aversive response in *Drosophila melanogaster* and *D. suzukii* [32]. This suggests that the Orco/Or mediated pathway likely is a conserved mechanism underlying limonene repellency across insect species. Interestingly, similar to our observation with *Orco^−/−^* mosquitoes, knockout of *Orco* expression in *D. melanogaster* did not fully abolish limonene-evoked aversive response [32]. Based on our finding of reduced limonene repellency in the *TRPA1^−/−^* mosquitoes in this study, it is possible that activation of TRPA1 channels contributes to the Orco/Or-independent pathway, underlying limonene repellency against *Ae. aegypti*. However, we cannot rule out the possibility that TRPA1 channels are involved in a step downstream of the canonical Orco/Or-mediated repellency pathway; and another Orco- or TRPA1- independent mechanism underlies the residual repellency. Regardless, our study supports the accumulative evidence that repellency of essential oil components can be mediated by TRPA1-dependent pathways operating in parallel with or downstream of Or-mediated mechanism [27].

A previous study in *D. melanogaster* showed that while Orco (Or83b) contributes to fly repulsion to citronellal, the repellency was significantly reduced to a comparable degree in a TRPA1-deficient strain [27]. More recent studies show that *Ae. aegypti* TRPA1 proteins are involved in mediating contact repellency and/or antifeedant effects of naturally derived compounds, such as nepetalactone and cinnamodial, in an Orco-independent way [28,29]. In vitro studies have further demonstrated that citronellal oil, cinnamodial, and nepetalactone activate TRPA1 channels from *An. gambiae* and/or *Ae. aegypti* [27,28,29,33]. Apparently, sensitivities of TRPA1 splice variants to essential oil components could vary greatly in each mosquito species, and among species. For example, citronellal activates AgTRPA1A and D variants from *An. gambiae*, only the variant AaTRPA1C from *Ae. aegypti;* and both CpTRPA1A and C variants from *Culex pipiens pallens* [33]. Furthermore, AaTRPA1C and CpTRPA1C were hypersensitive to citronellal compared with *An. gambiae* TRPA1A and D [33]. Unfortunately, only the AgTRPA1A was further examined for sensitivity to other plant-derived compounds, including limonene which did not activate AgTRPA1A [33]. Future studies are needed to determine which *Ae. aegypti* TRPA1 variants can be activated by limonene to fully elucidate the mechanism of repellency involving TRPA1 channels in *Ae. aegypti*.

Although the TRPA1 channel is clearly involved in limonene repellency, our vapor toxicity data showed that the insecticidal action of limonene operates independently of TRPA1 (Figure 4). This finding is consistent with previous reports where TRPA1 proteins are involved in the antifeedant and repellent effects of cinnamodial, but not in its toxicity [29]. Intriguingly, limonene potentiates GABA-induced Cl^−^ currents of the Rdl receptor, which is a key target of several classes of insecticides, implicating the potential role of Rdl in limonene toxicity. A similar modulatory effect has been reported with other naturally occurring terpenoids, including carvacrol, pulegone, and nootkatone, all of which have been reported with both repellent and toxicity effects against different insect species [18,34,35,36]. Thymol is another potent monoterpenoid that is characterized as a positive allosteric modulator (PAM) of insect Rdl receptors, however in vitro electrophysiological study showed that thymol could induce agonistic effect when applied at high dosages [37]. GABA receptors are widely expressed in the insect nervous system and mediate inhibitory neurotransmission by allowing Cl^−^ influx upon GABA binding. Therefore, potentiation of Rdl GABA receptors by essential oil components including limonene may be a common mechanism of action of these plant terpenoids.

The potentiation of GABA-mediated inhibitory effect by these plant terpenoids is different from that of convectional Rdl-targeting natural toxins or insecticides, such as picrotoxin (PTX), dieldrin, and fipronil, which act as non-competitive antagonists by binding to a PTX-binding site on the Rdl receptors and block Cl^−^ current influx, leading to hyperexcitation of the insect nervous system [34]. Newer classes of Rdl-targeting insecticides, such as meta-diamides and isoxazolines, are also non-competitive antagonists that interact with Rdl proteins on non PTX-binding sites [38,39]. In contrast to the inhibitory modes of action of these commonly used synthetic insecticides targeting Rdl receptors, the majority of the plant-derived compounds identified to date act either as direct agonists of Rdl receptors or PAMs that potentiate GABA-induced Cl^−^ currents. Therefore, it is highly likely that these natural compounds interact with Rdl receptors via distinct binding sites. Future studies investigating the molecular interaction between limonene and *Ae. aegypti* Rdl receptors and identifying potential binding sites may help improve the application of limonene as an alternative insecticidal component.

To date, multiple plant-derived compounds with neurotoxic and spatial repellency effects have been shown to interact with more than one molecular target in insects. For instance, nootkatone acts on both Ors and Ir co-receptors to mediate spatial repellency in *Ae. aegypti*, while also targets the Rdl receptor to exert insecticidal and potentially contact repellent effects [18]. Carvacrol as an effective insect repellent and insecticide, not only interacts with the Rdl receptor as PAMs, but also functions as a non-competitive antagonist of insect nicotinic acetylcholine receptors in the cockroach and housefly [34,40]. In this study, we have shown limonene as an additional plant-derived compound targeting multiple ion channels in insects to exert repellency and/or neurotoxic effect against mosquitoes. Plant secondary metabolites have evolved as chemical defenses against herbivores including insects, and the ability to act on multiple ion channels may enhance their defensive efficacy. Our findings provide valuable information to further advance understanding of the long-term co-evolutionary arms race between plants and insects and offer insights useful for the development of plant-based insecticides/repellents.

## 4. Materials and Methods

### 4.1. Mosquito Culture and Chemicals

Three *Ae. aegypti* mosquito lines were used in this study: *Orco^−/−^*, *TRPA1^−/−^* and Orlando. *Orco^−/−^* is a mutant line where an *Or* co-receptor gene (*orco^16^*) was mutated resulting in impaired *Or*s-mediated olfactory responses (BEI, Resources the National Institute of Allergy and Infectious Diseases, NIH [23]. *TRPA1^−/−^* is a mutant line where a *TRPA1* gene (*TRPA1^ECFP1/ECFP2^*) was mutated to generate a thermotaxis-impaired line (kindly provided by L. Vosshall, Rockefeller University; [30]. Orlando (kindly provided by L. Vosshall, Rockefeller University, New York, NY, USA) is a wildtype line from which *Orco^−/−^* and *TRPA1^−/−^* lines were generated.

Mosquitoes were maintained in an environmental growth chamber at the Phytotron (Department of Biology, Duke University) at 26 ± 1 °C, with 60–65% relativity humidity, and 12:12 h light: dark photoperiod. Mosquito larvae were hatched in a plastic container (1.57 × 0.77 × 1.41; Pactiv, Lake Forest, IL, USA) filled with reverse osmosis (RO) water and provided with liver powder (CurEase, McEwen, TN, USA) daily until pupation. Pupae were collected into 60 mL plastic cups (Dart, Mason, MI, USA) filled with RO water and transferred into 30 × 30 × 30 cm mesh cages before eclosion. Adult mosquitoes were fed 10% sucrose ad libitum after emergence. Females were blood-fed at least 4 days after emergence with defibrinated sheep blood (Colorado Serum Company, Denver, CO, USA) and then provided with filter paper (Whatman™, Cytiva, Wilmington, DE, USA) soaked in distilled water for oviposition. Gamma-amino butyric acid (GABA; CAS # 56-12-2, purity ≥ 99%) and (R)-(+)-limonene (CAS # 5989-27-5, purity 97%) used in this study were purchased from Sigma-Aldrich Corporation (St. Louis, MO, USA). Mated, non-blood fed adult female mosquitoes 4–9 days post emergence were used in all behavioral assays.

### 4.2. Hand-in-Cage Bioassay

To assess the non-contact spatial repellency by limonene against *Ae. aegypti*, we conducted a hand-in-cage assay as previously described [17]. Thirty to forty mosquitoes were randomly selected using a mouth aspirator and carefully released into an experimental cage 18 h before the assay. Limonene was pre-diluted in acetone to application of 500 µL at concentrations of 10^3^ and 10^4^ ppm. Immediately before the assay, the bottom net was treated with 500 μL of acetone alone, or limonene diluted in acetone, achieving doses of 12 µg/cm^2^ and 120 µg/cm^2^, respectively. After acetone fully evaporated, the treated bottom net and untreated top net were assembled into the modified glove. A tester wearing the modified glove placed their hand into the mosquito cage to initiate the assay. Mosquito landings from the second to the fifth minute were counted in real time for each cage. For each cage, solvent control was tested first, followed by limonene treatment. The time interval between control and treatment was at least 1.5 h, allowing the mosquitoes time to fully recover from the solvent trial. Repellency index was determined for each cage using the following equation: percentage of repellency = [1 − (cumulative number of landings on the window of treatment/cumulative number of landings on the window of solvent treatment)] × 100. For each limonene concentration, at least five replicates were conducted. A non-parametric Mann–Whiteney test was carried out to compare limonene repellency between control and limonene treated groups. SigmaPlot (version 15; Grafiti LLC., Palo Alto, CA, USA) was used for statistical analysis and GraphPad Prism (version 9.5.1; GraphPad Software Inc., La Jolla, CA, USA) was used for figure generation.

### 4.3. Spatial Repellency Tube Assay

A two-choice spatial repellency assay lacking host cues was modified from previously published studies and conducted to quantify the aversion behavior of female mosquitoes against limonene [41,42]. The setup consisted of a 30 × 5 cm glass tube (WGV^®^ International, El Monte, CA, USA) and two modified polystyrene Petri dishes (6 × 1.5 cm; Fisher Scientific^®^ International, Inc., Hampton, NH, USA) placed in both ends of the tube (Figure 3A). The bottom of each modified Petri dish was replaced by a 4 cm polyester net (Shason Textile Inc., Los Angeles, CA, USA, part number: WS-B532-111, Walmart, Bentonville, AR, USA, # 567948282, white). The net prevents mosquitoes from contacting the repellent and escaping the glass tube. Each Petri dish lid was drilled with two holes (diameter: ~0.5 cm) to permit air flow. The edge of each Petri dish base was wrapped with parafilm membranes to seal to the glass cylinder, and the attachment was further secured using clear polyester tapes.

Before initiating the assay, one modified Petri dish was placed at one end of the glass tube using polyester tape. Twenty mosquitoes were knocked down by CO_2_ and carefully transferred to center of the glass tube with a long handle modified from a serological pipette. The second modified Petri dish was immediately attached to the opposite end of the tube. Mosquitoes were allowed to recover and acclimate for at least 20 min before the assay began, during which they distributed themselves freely throughout the tube, resting without any apparent spatial bias and occasionally walking or flying. In an adjacent room, 5.15 μL of limonene was pipetted onto the center of a 50 mm diameter filter paper (Whatman™) for a 4.2 mg administration. The treated filter paper was immediately placed into the Petri dish on one side of the tube, and an untreated filter paper was simultaneously placed into the opposite Petri dish. To prevent mosquitoes from directly contacting limonene, filter papers were cautiously positioned to ensure they leaned against the Petri dish lids but not towards the polyester net (~1.0 cm away from the net). The tubes were placed on a white Styrofoam board with black line drawn in the middle; double sided tapes were glued to the board to keep the tubes from rolling. The location of each tube on the Styrofoam board and the side of the tube containing the repellent-treated filter were randomized. Mosquitoes were observed at 5 min post exposure, and a video was manually taken for each tube to record the number of mosquitoes on either side of the tube. The percentage of repellency of each tube was calculated using the following formula: (#_control_ − #_treated_)/(#_control_ + #_treated_ + #_unsetteled_) × 100, where #_control_ and #_treated_ represent the numbers of mosquitoes on the control side and limonene-treated side of the tube, respectively, and #_unsetteled_ represents the number of mosquitoes that moved across the middle line of the Styrofoam board. The repellency was corrected by the exact number of mosquitoes inside each tube. Two mutant lines, *Orco^−/−^* and *TRPA1^−/−^*, were tested in this bioassay. Each mutant line was subjected to three experimental trials, with each trial consisting of 6 replicates (i.e., tubes) for the mutant line per se and additional six replicates for the wildtype Orlando. Student’s *t*-tests were carried out to compare limonene repellency between mutant and wildtype lines. SigmaPlot was used for statistical analysis and GraphPad Prism was used for figure generation.

### 4.4. Vapor Toxicity Assay

A vapor toxicity assay modified from Zhu et al. [43] was conducted to evaluate limonene insecticidal effect against wildtype Orlando and *TRPA1^−/−^* female *Ae. aegypti* (Supplementary Figure S4). Eighteen to twenty-one female mosquitoes were sedated briefly on ice and carefully transferred into a modified Petri dish (diameter: 9 cm; height: 1.4 cm). 5.15 µL and 51.5 µL of 97% limonene was pipetted onto the center of a 70 mm diameter filter paper (Whatman™) for 4.2 mg and 42.1 mg administrations, respectively. The treated filter paper was placed on top of an untreated filter paper in a second Petri dish base to prevent limonene from damaging the plastic. The mosquito-containing Petri dish was immediately placed over the second Petri dish base and sealed together using parafilm tapes. Negative control groups were included for each trial, where two pieces of untreated filter papers were placed inside. No abnormal behavior or mortality of mosquitoes were observed in the negative control group over the experimental course. The number of knocked down mosquitoes was monitored at 0.5, 1, and 2 h post exposure. A repeated-measures two-way ANOVA was conducted in SigmaPlot to determine the effects of time and genotype on the percentages of knockdown. GraphPad Prism was used for figure generation.

### 4.5. Electrophysiology Assay

#### 4.5.1. cRNA Synthesis, Oocyte Preparation, and cRNA Injection

A Rdl clone and a sodium channel clone from female *Ae. aegypti* were used in this study: *AaegRdl1-1* [18] and *AaNa_v_1-1* [44]. In vitro transcription was performed using a commercially available kit (mMessage mMachine T7 Transcription Kit; Thermo Fisher Scientific, Waltham, MA, USA) and synthesized cRNA was stored at −80 °C until further use. *Xenopus laevis* oocytes were obtained from Xenopus1 corp. (Dexter, MI, USA) in the form of dissociated ovaries. Mature oocytes of stage V-VI were selected to remove their follicle cells and follicle membranes. Defolliculated oocytes were injected 1–2 ng of cRNA and incubated at 18 °C in culture solution until ready for testing.

#### 4.5.2. Two Electrode Voltage Clamp

Recordings of *Xenopus* oocytes were conducted using an oocyte clamp instrument OC725-C amplifier (Warner Instrument Cor., Hamden, CT, USA), a Digidata 1200A interface (Axon Instrument, Foster City, CA, USA), and a Clampex software (version 10.7; Molecular Devices, San Jose, CA, USA).

For oocytes expressing *AaNa_v_1-1*, oocytes were clamped to −120 mV and the protocols were conducted as previously described [45]. For oocytes expressing *AaegRdl1-1*, recording components were set up in a 3D-printed polylactic acid chamber and conducted at room temperature. The oocyte chamber was constantly perfused with bath saline. A solenoid pinch valve was installed on the tubing near the output of the syringe filled with bath saline, and turning off the valve stopped the bath saline from flowing into the chamber. Another tubing was used to direct the waste saline out of the chamber into a container placed beneath the recording chamber. A modified U-tube system was utilized to accomplish a precise and instant release of agonist and/or chemicals towards the oocytes [46]. A second solenoid pinch valve was installed downstream of the U-tube delivery system. When the valve was off, the vacuum was discontinued and pressure inside the tube resulted an instant release of U-tube saline at oocyte through the pin hole pointing at the oocyte.

Recording protocols were set up as follows. Each recording sweep lasted for 120 s with a sampling rate per signal set at 100 Hz. Oocytes were clamped at −80 mV and only the ones with moderate and stable leak currents below 200 nA were used for recordings. Both pinch valves were controlled by a TTL triggered valve controller (VC-6, Warner Instrument Cor.) and digitally closed for a duration of 5 s at 10 s post the start of each recording sweep, which stopped the bath flow from syringe and initiated the U-tube application of agonist and/or chemicals towards the oocyte. Once the valves were turned back on, the bath flow continued flowing into the chamber and effectively washed off the agonists and/or chemicals inside, while the U-tube saline was draining into the vacuum without leaking into the oocyte chamber. To better visualize the range and angle of agonist and/or drug application at the oocyte, recording saline containing 1% fluorescein sodium salt (Sigma-Aldrich) was used for preliminary pulses, after which the U-tube system was thoroughly rinsed with recording saline before the recording started.

#### 4.5.3. Data Collection and Statistical Analysis

To generate a GABA concentration response curve of *AaegRdl1-1*, each concentration of GABA ranging from 10 to 1000 μM was tested until a maximum response plateau was achieved. To undermine the effect of limonene, 100 μM limonene was added along with the application of each concentration of GABA to generate a GABA response curve in the presence of limonene. Response curves of each individual cell were fitted into a dosage response curve in GraphPad Prism. EC_50_ values with or without the presence of limonene were calculated for each individual cell and the averages were compared using a Student’s *t*-test. A global response curve fit was performed using data collected from all five oocytes and the curve was plotted using GraphPad Prism.

Once GABA response curves with and without limonene co-application were generated, the GABA concentration that induced the biggest difference in peak current amplitude (i.e., 100 μM GABA) was chosen to generate a limonene response curve with the limonene concentration ranging from 1 to 100 µM. Solutions were manually switched during recordings to ensure that both the bath saline and U-tube saline contained the same concentration of limonene. Limonene response curves were fitted into a dosage response curve for each individual oocyte and EC_50_ values were calculated. A global response curve was generated based on the pooled data from five oocytes and plotted using GraphPad Prism.

## 5. Conclusions

Our study is the first to reveal that multiple ion channels and receptors are involved in the repellent and/or insecticidal actions of limonene against mosquitoes. While limonene repellency appears to be mediated through both Or-dependent and TRPA1-involved pathways, its toxicity likely results from prolonged inhibitory synaptic transmission caused by enhanced GABA-induced chloride influx through the Rdl receptor. Taken together, our results offer valuable insights for employing limonene as a naturally derived insect repellent and insecticide as well as using limonene as a model for investigating neuronal mechanisms underlying mosquito behavioral and electrophysiological responses.

## Data Availability

The original contributions presented in this study are included in the article/Supplementary Materials. Further inquiries can be directed to the corresponding authors.

## Supplementary Materials

The following supporting information can be downloaded at: https://www.mdpi.com/article/10.3390/ijms27010416/s1.
