# Supplementary material for: Involvement of Multiple Ion Channels and Receptors in Mediating the Insecticidal and Repellent Actions of Limonene
Source: Int J Mol Sci. 2025 Dec 30;27(1):416. doi: 10.3390/ijms27010416 (PMC12787074; doi:10.3390/ijms27010416)
Supplement: Supplementary file 1 [file ijms-27-00416-s001.zip › ijms-4046677-supplementary.pdf]

Supplemental material

Involvement of multiple ion channels and receptors in mediating the insecticidal and repellent actions of limonene

Yuan Li, Wilson Valbon, Felipe Andreazza*, and Ke Dong*

Department of Biology, Duke University, Durham, NC, 27708, USA

*ke.dong@duke.edu and felipe.andreazza@duke.edu; Duke University, 130 Science Drive, Durham, NC 27708; Phone: 919-660-3186.

Contents:

Supplemental Figures 1 to 4.

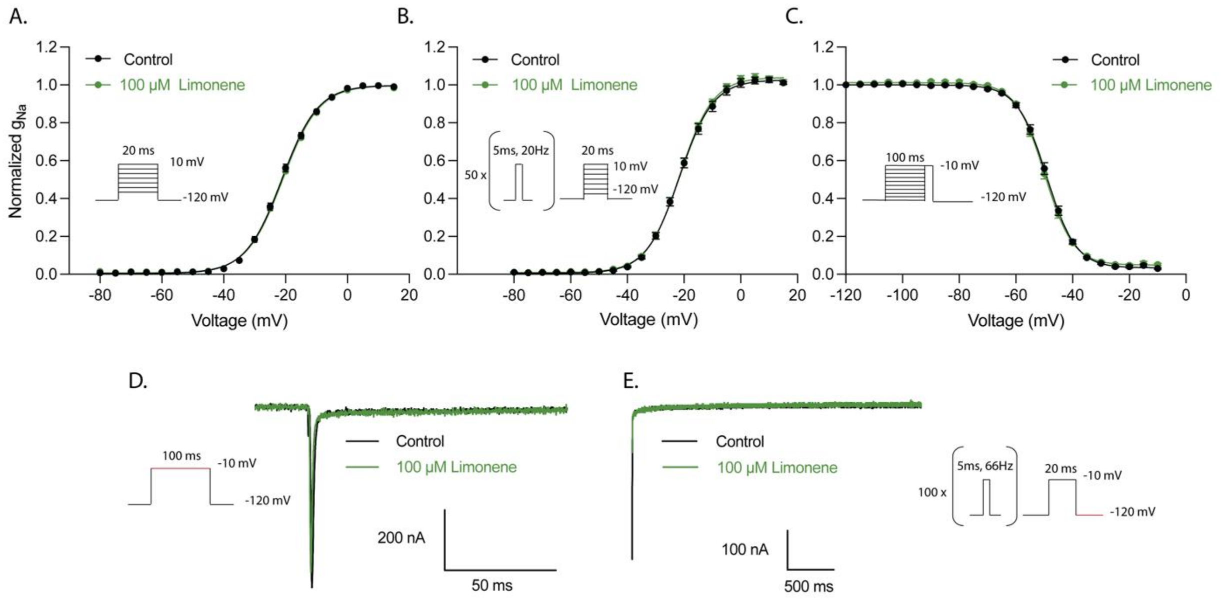

Figure S1. Voltage-gated sodium channel is not the molecular target of limonene *in vitro*. A). Voltage-dependence of channel activation. B). Voltage-dependence of channel activation after a train of 50 times 5 ms pre-pulses to +50 mV at 20 Hz. C). Voltage-dependence of channel inactivation. D). Representative sodium current traces of protocol testing non-inactivation current. Limonene did not elicit non-inactivation current. E). Representative sodium current traces of protocol testing tail current. Limonene did not elicit tail current.

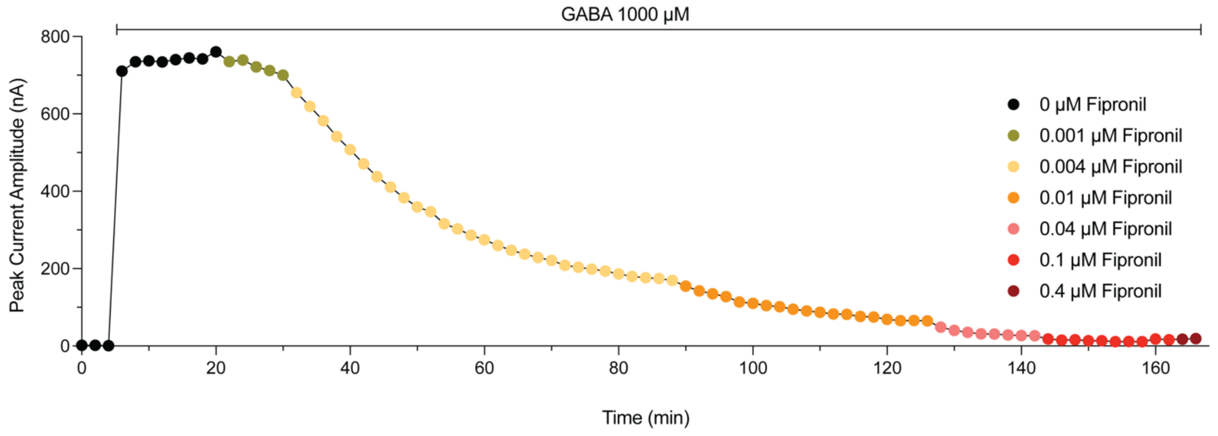

Figure S2. Peak current amplitude of an Rdl-expressed *Xenopus* oocyte in response to Fipronil concentrations ranging from 0 to 0.4 µM. Co-application of 1000 µM GABA in the U-tube initiated at 6 min. Each treatment was repeated for at least three times. Oocyte was clamped at -80 mV.

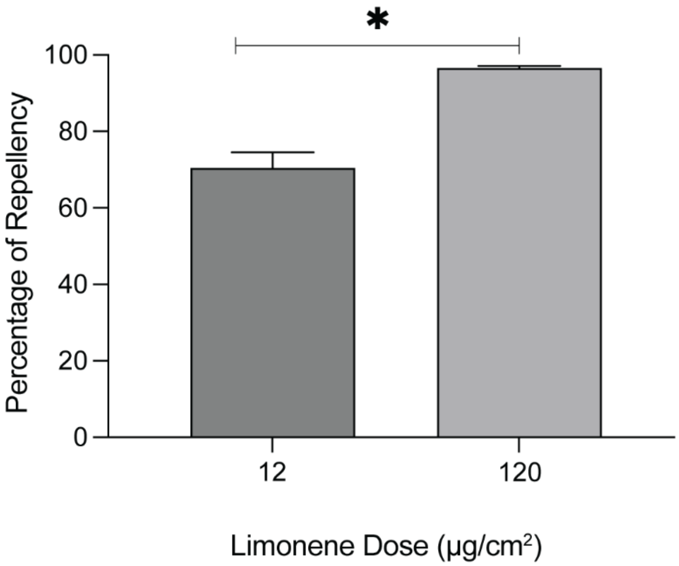

Figure S3. A). Percentages of repellency of wildtype Orlando against 12 µg/cm² (n = 5) and 120 µg/cm² (n = 7) limonene application in a hand-in-cage bioassay. Asterisk indicates significant difference between two treatments as determined by Mann-Whiteney test ($p < 0.05$).

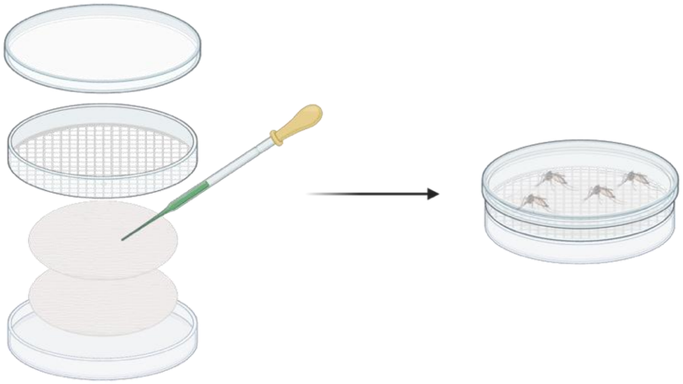

Figure S4. Experimental setup of vapor toxicity assay. Illustration created in BioRender.
